# Detection of Retinal Hemangioblastomas in von Hippel–Lindau Disease Using Three-Dimensional Arterial Spin Labeling MR Imaging at 3T

**DOI:** 10.2463/mrms.ci.2016-0030

**Published:** 2016-07-05

**Authors:** Jun-ichi Nomura, Takaaki Beppu, Makoto Sasaki, Shunrou Fujiwara, Kuniaki Ogasawara

**Affiliations:** 1 Department of Neurosurgery, Iwate Medical University, 19-1 Uchimaru, Morioka, Iwate 020-8505, Japan; 2 Division of Ultrahigh Field MRI, Institute for Biomedical Sciences, Iwate Medical University

**Keywords:** ASL, hemangioblastoma, von Hippel–Lindau disease, orbital lesion

Approximately, 30% of hemangioblastomas of the central nervous system (CNS) are caused by von Hippel–Lindau disease (VHL).^1^ Patients are diagnosed with VHL if they have two or more CNS hemangioblastomas (including retinal hemangioblastomas) or one CNS hemangioblastoma and a visceral tumor (with the exception of epididymal and renal cysts, which are frequent in the general population).^2^ Since hemangioblastomas are pathologically hypervascular due to the overexpression of vascular endothelial growth factors, their capillary density is found to correlate with the signal intensity of magnetic resonance (MR) perfusion imaging, thereby indicating cerebral blood flow. In the case of a patient with VHL, we were able to detect retinal hemangioblastomas as well as cerebellar lesions by non-invasive three-dimensional arterial spin labeling (3D-ASL).

The patient was a 38-year-old woman who was diagnosed with VHL, 8 years ago. A mass was detected in the left cerebellar hemisphere, and she underwent a craniotomy for surgical resection of the mass, which was histopathologically confirmed as a hemangioblastoma. Two years later, MR imaging indicated that there was no recurrence of CNS hemangioblastomas or any new lesions. However, 6 years later, a T_1_-weighted image (T_1_WI) with gadolinium (Gd) contrast agent revealed new masses in the right cerebellar hemisphere and medulla oblongata.

During the same MR imaging scan that revealed these findings, we also performed 3D-ASL (3D fast spin-echo acquisition with spiral readout, pseudo-continuous ASL (pCASL); repetition time/effective echo time, 4590/10.5 ms; field of view, 240 × 240 mm^2^; labeling time, 1450 ms; post-labeling delay, 1525 ms; slice thickness, 4.0 mm; number of slices, 32; and number of excitations, 2) using a 3-Tesla MR imaging (3-TMRI) system (Discovery MR750, GE Healthcare, Milwaukee, WI, USA) with an eight-channel phased array coil. The two mass lesions showed increased blood flow on 3D-ASL. In addition to these lesions, the 3D-ASL clearly demonstrated a region of increased blood flow in the left retina (Fig. 1A), which was barely identified on Gd-T_1_WI (Fig. 1B). Six months later, the two lesions in the right cerebellar hemisphere and medulla oblongata showed no enlargement on MR imaging. The masses may have been hemangioblastomas, although histopathological examination was not performed for confirmation. Fundoscopy revealed that the lesion in the left retina was a hemangioblastoma (Fig. 1C).

To the best of our knowledge, this is the first report to describe the value of 3D-ASL in detecting retinal hemangioblastomas. ASL using 2D-echo planar imaging may result in image distortion, due to air in the paranasal sinuses, and therefore, cannot be used for the detection of masses in the orbital region.^3^ In this case, we used pCASL with 3D fast spin-echo imaging with spiral readout to cover the entire head and decrease distortion, in addition to the high magnetic field at 3T for a higher signal-to-noise ratio than that at 1.5T. We believe that 3D-ASL at 3-TMRI may allow the detection of retinal hemangioblastomas. The conventional Gd-T_1_WI allowed limited detection of retinal hemangioblastomas, while 3D-ASL clearly demonstrated the lesion, which indicated increased blood flow. These findings suggest that 3D-ASL can aid in the identification of retinal hemangioblastomas with high sensitivity in patients with VHL.

## Figures and Tables

**Fig 1.**
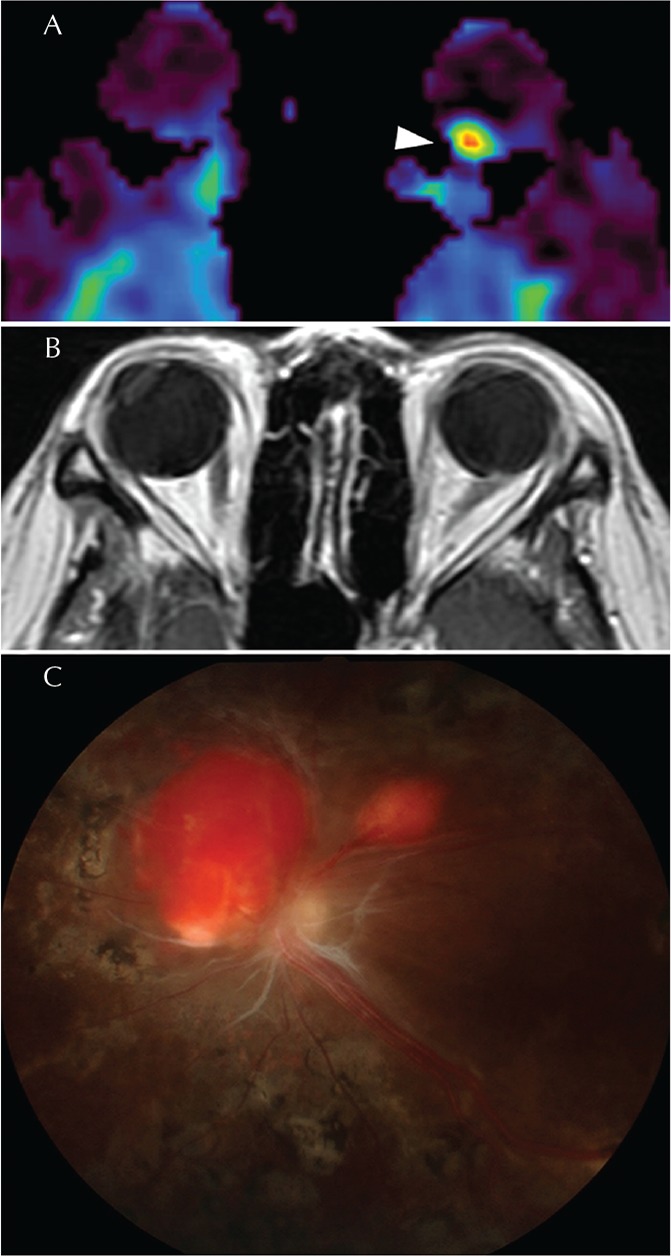
A three-dimensional arterial spin labeling (3D-ASL) image at the orbital level clearly indicates increased blood flow in the left retina (**A**, arrowhead), while a gadolinium-enhanced T_1_-weighted image (GD-T_1_WI) could only minimally indicate the abnormality in the same region (**B**). Fundoscopy of the left eye confirms retinal hemangioblastomas (**C**).
